# Periodontal diseases and potential risk factors in Egyptian adult population—Results from a national cross-sectional study

**DOI:** 10.1371/journal.pone.0258958

**Published:** 2021-11-03

**Authors:** Reham Khaled Abou El Fadl, Mona Ahmed Abdel Fattah, Muhammad Ahmed Helmi, Mariem Osama Wassel, Amira Saad Badran, Huda Ahmed Amin Elgendi, Mona Ezz Eldien Allam, Ahmed Gamal Mokhtar, Mostafa Saad Eldin, Eslam Ahmed Yahia Ibrahim, Bahaaeldeen M. Elgarba, Mustafa Mehlis

**Affiliations:** 1 Pediatric Dentistry and Dental Public Health Department, Faculty of Dentistry, Ain Shams University, Cairo, Egypt; 2 Cairo Dental Research Center, Ministry of Health & Population, Cairo, Egypt; 3 Institute of Tropical Medicine, and International Health, Charité–Universitätsmedizin Berlin, Berlin, Germany; 4 Central Administration of Dentistry, Ministry of Health & Population, Alexandrina, Egypt; 5 Faculty of Dentistry, Sinai University, Kantata, Egypt; 6 Ministry of Health & Population, Cairo, Egypt; 7 Faculty of Dentistry, Modern University for Technology & Information, Cairo, Egypt; 8 Faculty of Dentistry, Tanta University, Tanta, Egypt; 9 PennyPot Dental Practice Ltd., Ashford, Kent, United Kingdom; Griffith University, AUSTRALIA

## Abstract

**Background:**

Despite the interdependence of general and periodontal health, there is paucity of national representative data on the prevalence of periodontal diseases and their associated risk factors in Egyptian population. This cross-sectional study, thus, aimed to assess the prevalence of periodontitis and tooth loss among Egyptian adults and investigate the association between potential risk factors and periodontal diseases.

**Methods:**

A total of 5,954 adults aged ≥ 20 years were included in this study as a subsample from Egypt’s national oral health survey. Periodontitis was diagnosed with Community Periodontal Index ‘CPI’ scores ≥3 and tooth loss not due to caries was included in the analysis. Socio-demographic data and information on behavioral factors and history of diabetes were gathered in a face-to-face interview. Logistic regression was done to interpret the impact of potential predictors on the incidence of the two selected outcome variables.

**Results:**

The overall prevalence of periodontitis was 26% and regression analysis revealed that higher odds of periodontitis existed among illiterate participants (OR = 1.74; 95% CI: 1.40–2.17), smokers (OR = 1.93; 95% CI: 1.69–2.20) and rural residents (OR = 1.16; 95% CI: 1.03–1.30). On the other hand, old age, frequency of dental attendance and history of diabetes were the main predictive factors for tooth loss.

**Conclusions:**

Among Egyptian adults, periodontal diseases were strongly associated with a multitude of modifiable and non-modifiable risk factors and inequalities in distribution of periodontal treatment needs were determined mainly by age, gender, level of education and residency location.

## 1. Introduction

Periodontal disease is one of the two most major oral conditions contributing to the global burden of chronic diseases [1]. It has been reported that, worldwide, over 5 million people suffer from severe periodontitis with a 3.8% increase in the number of all age-disability-adjusted life-years (DALYs) for periodontal disease from 1990 to 2016 which renders it a major public health problem [2–4]. Generally, the prevalence and severity of periodontal disease follow a social gradient where disadvantaged populations with fewer years of education are more commonly affected [5]. Besides, it is widely known that periodontitis increases with age and its economic burden has been on the rise lately due to the noticeably ageing population worldwide [6].

One major consequence of periodontitis in severe cases is tooth loss which subsequently leads to tooth migration, drifting, masticatory dysfunction and ultimately edentulism [7]. Consequently, periodontal problems can adversely affect people’s self-esteem and quality of life from emotional, psychological, social, and functional perspectives [8]. Furthermore, along with other oral conditions, periodontitis, shares common risk factors with major non-communicable diseases such as cardiovascular disease, cancer, and diabetes. Among those are non-modifiable risk factors including genetically determined hyper-inflammatory polymorphisms, age, and gender as well as behavioral factors, such as tobacco and alcohol consumption, poor dietary habits, and psychosocial factors [4, 9–11].

Acknowledging that periodontal problems are preventable, there is a strong call for up scaling global action to implement effective diagnostic, preventive, and therapeutic strategies to reduce periodontal disease burden at individual, national and global levels [12]. Egypt is a North African densely populated, low-middle income country with a total population of over 100 million people of which 57.3% reside in rural areas and out-of-pocket payments account for over 60% of total health expenditures [13]. Although there is lack of nationwide comprehensive information about oral health status of Egyptian population, in few cross-sectional studies conducted at province level, high prevalence rates of different periodontal problems ranging from 69.4 to 89.8% were reported [14, 15].

Since no country-wide oral health assessment in Egypt has been done since early 90s and little is known about the prevalence and severity of oral conditions, a national oral health survey was conducted in 2013–2014 to establish baseline data on oral health status and inform planning policies and strategies needed for oral disease prevention and health promotion at national level [16].

Thus, the purpose of the current study was to determine the prevalence and distribution of periodontal problems among Egyptian dentate adults and identify potential risk factors associated with periodontitis and tooth loss using data from Egypt’s national oral health survey (2013–2014) provided by the Ministry of Health and Population (MOHP).

## 2. Methods

Data used in the present study is from a population‐based face to face cross‐sectional survey conducted under the auspices of Central Administration of Dentistry, Egyptian MOHP [16]. An ethical approval *(protocol no*. *15-2013/4)* was granted by the Research Ethical Committee, Egyptian MOHP, Central Directorate for Research and Health Development on May 7^th^, 2013.

The sample size was measured according to Egypt’s Census dataset of 2013, provided by the Egyptian Central Agency for Public Mobilization and Statistics (CAPMAS). A total of 10,144 participants were enrolled in a population-based survey, to give a 95% confidence level, a sample power of 99.32% with a marginal error of 0.68%. Given the low prevalence of periodontal problems among children and adolescents, periodontal assessment was performed only for participants aged 20 and older. The analytical sample for the current study, thus, included adults aged ≥ 20 years with complete information on all the study variables (n = 5,954 participants).

Data collection started at the end of September 2013 and was completed in mid-May 2014. Prior to enrollment, each participant was informed about the survey purpose, its benefits for the Egyptian population, the nature of undertaken procedures, any possible discomfort as well as data confidentiality and the right to withdraw during data collection. All eligible participants signed informed consents in simple Arabic language except illiterate participants whose fingerprints were used instead of signatures. This survey was conducted according to the principles of the Declaration of Helsinki on experimentation pertaining to human subjects (version 2013).

The national survey adopted a stratified multistage clustered random sampling approach where sampling was done in 3 stages based on the administrative divisions of the country. Geographically, Egypt is divided into 4 regions; *Lower Egypt*, *Upper Egypt*, *Desert/Frontier and Civilized* regions with a total of 27 governorates distributed among those regions. Each governorate is further subdivided into urban and rural areas except for five *civilized* governorates which include only urban areas. The survey sample was taken from 26 governorates and one governorate *‘North Sinai’* was excluded for security reasons. The primary sampling units were randomly selected from a sampling frame obtained from CAPMAS. The frame comprised a list of all localities in each governorate which are known as *kism* in urban areas and *markaz* in rural areas. This was followed by the second level cluster which included 160 gathering points; mainly at primary healthcare centers, public hospitals, few households, and workplaces. In the final stage of sampling, individuals were systematically randomly selected using lists of patients attending the health facility on the day of recruitment. All Egyptian dentulous males and females in all age groups were eligible for the study. Only individuals seeking dental care *per se* and those who withheld consent or had no teeth at the time of dental examination *(edentulous)* were excluded.

The outcomes for this study were (i) the presence of periodontitis and (ii) tooth loss not due to dental caries. Participants were asked about the reason for tooth loss and were considered to have periodontitis when they scored 3 or more on the WHO Community Periodontal Index (CPI), indicating that at least more than one surface of any index tooth had a 4-mm pocket or deeper.

Health behaviors analyzed in the current study included frequency of tooth brushing, smoking and dental attendance. Frequency of brushing was assessed by reporting the number of times respondents cleaned their teeth daily. Smoking status was determined by a three‐category variable identifying current smokers, former smokers and non-smokers and dental attendance was measured by assessing time elapsed since last dental visit.

Potential confounders included age, gender, and geographic location *(urban/rural)*. Level of education was used as an indicator of socioeconomic status (SES) and was categorized into three categories *(illiterate*, *high school education or less or 2 years academy & some college or above)*. Data was also collected on medical history of diabetes mellitus and participants were assigned to one of two categories: normal or diabetic.

Data on participants’ characteristics and potential risk factors was obtained using a structured questionnaire through interviews and dental examination was carried out in accordance with the World Health Organization (WHO) basic methods for oral health surveys [17].

All dental examinations were conducted either in a clinic or in the field using plane mouth mirrors, WHO CPI probes and lightweight portable light sources and all participants had to face away from natural light to avoid any variation in illumination. The whole mouth was divided into six sextants and periodontal health status was assessed on a scale ranging from 0 to 4 using the three components of the community periodontal index (CPI); bleeding on probing *(CPI 1)*, presence of calculus *(CPI 2)* and pocket formation 4- to 5-mm deep *(CPI 3)*, or a 6-mm or deeper pocket *(CPI 4)*. CPI score 0 was given to participants with healthy gums and sextants were examined when they included at least two functioning teeth [18].

Ten index teeth; two molars in each posterior sextant as well as upper right and lower left incisors were examined by moving the WHO CPI probe slowly from the distal to the mesial surfaces along both the buccal and lingual sulci of each of the index teeth. No replacement tooth was examined if an index tooth was missing, however when all index teeth were missing from a sextant, all present teeth in that sextant were examined instead. The highest tooth score was, then, recorded as the score for a sextant and the highest CPI score for all examined teeth was recorded as the CPI score for each participant [17].

Oral health examination and interviews were performed by a total of 20 dentists; 5 principal investigators and 15 co-investigators recruited from the Egyptian MOHP and Faculty of Dentistry, Ain Shams University. All investigators attended a 5-day workshop for training on survey procedures and calibration was repeated across the duration of data collection to ensure consistency. Kappa statistic and Cronbach’s alpha reliability coefficients were used to assess intra and inter-observer agreement regarding CPI scores and pocket depth (PD) measurements respectively. Agreement values are interpreted as: 0–0.2: weak agreement; 0.2–0.4: fair, 0.4–0.6: moderate, 0.6–0.8: good, 0.8–0.99: very good and a value of 1 indicates perfect agreement [19]. As regards CPI scores; there was very good intra-and inter-observer agreement (Kappa = 0.858) and (Kappa = 0.813), respectively. On the other hand, for PD measurements; there was very good intra-observer agreement (Cronbach’s alpha = 0.854) and good inter-observer agreement (Cronbach’s alpha = 0.779) [20].

Unweighted data analysis was performed using IBM^®^ SPSS^®^ (SPSS Inc., IBM Corporation, NY, USA) Statistics Version 19 for Windows. Descriptive statistics was expressed for continuous variables by mean (M), standard deviation (SD) and unpaired t-test or one way ANOVA were used to compare between mean values. Frequency distribution was expressed by number and percentage for categorical variable and Chi-square test, or its subsidiaries were used to test statistical significance of different associations between independent variables and categorical clinical outcomes.

Odds ratio (OR) and 95% confidence interval were calculated using logistic regression to interpret the impact each of the assumed predictors such as behavioral factors, level of education, age, and gender on the incidence of the two selected outcome variables *periodontitis and tooth loss*. Multivariate logistic regression was performed to identify the independent variables among the potential risk factors which can best predict incidence of periodontitis. For all statistical tests, a *p-value* of less than 0.05 was considered statistically significant.

## 3. Results

From an initial eligible sample of 6216 adults aged 20 and above, 262 were excluded due to missing data on one or more of study variables. Among the 5954 participants, 51.28% (3053) were females, 53.60% resided in urban areas and ages ranged from 20 to 85 years old; with a mean age 38.55 years (SD = 11.73). The proportions of participants who were illiterate, finished high school or less and 2 years academy or college and above were 9.08%, 60.61% and 30.31% respectively. Most of the study sample reported visiting the dentist within the past six months (44.21%), 70% had never smoked, 35.64% reported never brushing their teeth and only 9.93% brushed at least twice daily.

***Table 1*** shows the distribution of gingival and periodontal problems among socio-demographic variables, behavioral factors and history of diabetes. Overall, 26% of the Egyptian adults had periodontitis, 3.2% had a severe form of periodontitis, and only 7% had healthy gums with no bleeding on probing, calculus, or pocket formation. Whereas bleeding and calculus formation were more common among females, periodontal destruction was seen more frequently in males, less educated participants, and those ≥45 years.

**Table 1:** Socio-demographic characteristics of study participants and the distribution of the periodontal outcomes within the study variables.

Characteristic			Periodontal Outcomes N (%)			
	Total no. of participants	Tooth Count # (Mean ± SD)	Bleeding	Calculus	Pocket (4-5mm)	Pocket ≥6mm
**Total**		**25.06 ± 4.03**	**397 (6.67%)**	**3672 (61.67%)**	**1368 (22.98%)**	**186 (3.12%)**
**Age**						
20–34 years	2490 (41.8%)	26.94 ± 1.89	248 (9.96%)	1574 (63.21%)	420 (16.87%)	31 (1.24%)
35–44 years	1529 (25.7%)	25.13 ± 3.27	75 (4.91%)	983 (64.29%)	374 (24.46%)	43 (2.81%)
45–59 years	1736 (29.2%)	22.99 ± 4.74	67 (3.86%)	1004 (57.83%)	516 (29.72%)	95 (5.47%)
≥ 60 years	199 (3.3%)	18.98 ± 6.74	7 (3.52%)	111 (55.78%)	58 (29.15%)	17 (8.54%)
*p-value* *		***< 0*.*001***	***< 0*.*001***	***< 0*.*001***	***< 0*.*001***	***< 0*.*001***
**Gender**						
Male	2901 (48.7%)	25.00 ± 4.20	135 (4.65%)	1747 (60.22%)	777 (26.78%)	123 (4.24%)
Female	3053 (51.3%)	25.11 ± 3.87	262 (8.58%)	1925 (63.05%)	591 (19.36%)	63 (2.06%)
*p-value*		*0*.*277*	***< 0*.*001***	***0*.*025***	***< 0*.*001***	***< 0*.*001***
**Residence location**			** **	** **	** **	** **
Urban	3167 (53.6%)	25.15 ± 3.93	233 (7.36%)	1916 (60.50%)	705 (22.26%)	83 (2.62%)
Rural	2742 (46.4%)	24.94 ± 4.16	163 (5.94%)	1727 (62.98%)	656 (23.92%)	103 (3.76%)
*p-value*		***0*.*045***	***0*.*030***	***0*.*050***	*0*.*130*	***0*.*013***
**Education**						
Illiterate	537 (9.1%)	23.61 ± 4.90	12(2.23%)	351 (65.36%)	140 (26.07%)	24 (4.47%)
High school or less	3585 (60.6%)	24.82 ± 4.22	220 (6.14%)	2207 (61.56%)	895 (24.97%)	122 (3.40%)
Two years academy college or above	1793 (30.3%)	25.97 ± 3.07	164 (9.15%)	1090 (60.79%)	321 (17.90%)	40 (2.23%)
*p-value*		***< 0*.*001***	***< 0*.*001***	*0*.*157*	***< 0*.*001***	***0*.*012***
**Tooth brushing**						
Never	2099 (35.6%)	24.30 ± 4.66	127 (6.05%)	1280 (60.98%)	556 (26.49%)	89 (4.24%)
2–3 times/month	517 (8.8%)	25.18 ± 3.82	20 (3.87%)	332 (64.22%)	143 (27.66%)	13 (2.51%)
Once/week	523 (8.9%)	25.50 ± 3.50	39 (7.46%)	333 (63.67%)	107 (20.46%)	24 (4.59%)
2–6 times/week	1288 (21.9%)	25.50 ± 3.51	90 (6.99%)	818 (63.51%)	285 (22.13%)	30 (2.33%)
Once/day	877 (14.9%)	25.57 ± 3.56	69 (7.87%)	544 (62.03%)	165 (18.81%)	15 (1.71%)
≥ twice/day	585 (9.9%)	25.65 ± 3.38	50 (8.55%)	325 (55.56%)	93 (15.90%)	13 (2.22%)
*p-value*		***< 0*.*001***	***0*.*017***	***0*.*016***	***< 0*.*001***	***< 0*.*001***
**Smoking status**						
Current smokers	1423 (24%)	24.66 ± 4.44	45 (3.16%)	829 (58.26%)	438 (30.78%)	69 (4.85%)
Non-smokers	377 (6.4%)	25.28 ± 3.80	328 (7.95%)	2606 (63.13%)	829 (20.08%)	92 (2.23%)
Former smokers	4128 (69.6%)	24.20 ± 4.55	21 (5.57%)	222 (58.89%)	97 (25.73%)	23 (6.10%)
*p-value*		***< 0*.*001***	***< 0*.*001***	***0*.*003***	***< 0*.*001***	***< 0*.*001***
**Dental attendance**						
Within past 6 months	1423 (18.4%)	24.56 ± 4.30	185 (7.13%)	1576 (60.73%)	595 (22.93%)	79 (3.04%)
6 months-1 year	377 (4.9%)	24.95 ± 3.81	63 (7.98%)	491 (62.23%)	166 (21.04%)	25 (3.17%)
1–2 years	4128 (53.4%)	24.50 ± 4.05	32 (5.34%)	372 (62.10%)	136 (22.70%)	29 (4.84%)
>2 years	1423 (18.4%)	25.23 ± 3.95	69 (5.32%)	830 (64.04%)	296 (22.84%)	34 (2.62%)
Never	377 (4.9%)	27.56 ± 1.73	46 (7.78%)	344 (58.21%)	156 (26.40%)	19 (3.21%)
*p-value*		***< 0*.*001***	***0*.*048***	*0*.*128*	*0*.*229*	*0*.*144*
**Diabetes**						
Normal	5544 (93.1%)	25.19 ± 3.90	378 (6.82%)	3446 (62.20%)	1251 (22.56%)	154 (2.77%)
Diabetic	410 (6.9%)	23.20 ± 5.14	19 (4.63%)	226 (55.12%)	117 (28.53%)	32 (7.80%)
*p-value*		***< 0*.*001***	*0*.*087*	***0*.*005***	***0*.*006***	***< 0*.*001***
*p-value*		*0*.*406*	***0*.*017***	***0*.*016***	***< 0*.*001***	***< 0*.*001***

*ANOVA and Chi-square tests were used to test significance of differences in mean values for no. of tooth count and periodontal outcomes respectively.

#Third molars were excluded when calculating tooth count.

The results of logistic regression analysis for factors associated with periodontitis are presented in **Table 2**. Males (OR = 1.65; 95% CI: 1.47–1.85), illiterate adults (OR = 1.74; 95% CI: 1.40–2.17) and smokers (OR = 1.93; 95% CI: 1.69–2.20) were at higher risk of developing periodontitis. Old age was also found to be a risk factor of periodontitis as the prevalence of CPI ≥3 was 37.69% among adults aged ≥60. Moreover, young adults had significantly lower odds of having periodontitis than those aged 35–44 years (OR = 0.37; 95% CI: 0.27–0.50).

**Table 2:** Logistic regression analysis showing the associations between periodontitis and potential risk factors.

*Variable*	Periodontitis CPI ≥ 3				
	N (%)	*p-value* *	*ß*	*p-value* **	Odds Ratio *(95% CI)*
**Age 20–34 yrs.**	451 (18.11%)	**<0.001**	-1.006	< 0.001	0.37(0.27–0.5)
**35–44 yrs.**	417 (27.27%)		-0.478	0.002	0.62(0.46–0.84)
**45–59 yrs.**	611 (35.20%)		-0.108	0.486	0.9(0.66–1.22)
**≥ 60 yrs.**	75 (37.69%)		**Reference**		
**Gender Male**	900 (31.02%)	**<0.001**	0.501	< 0.001	1.65(1.47–1.85)
**Female**	654 (21.42%)		**Reference**		
**Residence Rural**	759 (27.68%)	**0.015**	0.145	0.015	1.16(1.03–1.3)
**location Urban**	788 (24.88%)		**Reference**		
**Education Illiterate**	164 (30.54%)	**<0.001**	0.556	< 0.001	1.74(1.4–2.17)
**High school & less**	1017(28.37%)		0.452	< 0.001	1.57(1.37–1.80)
**2yrs. academy &**	361 (20.13%)		**Reference**		
**some college or above**					
**Teeth Never**	645 (30.73%)	**<0.001**	0.695	< 0.001	2 (1.59–2.52)
**brushing 2-3/ months**	156 (30.17%)		0.669	< 0.001	1.95 (1.47–2.59)
**Once a week**	131 (25.05%)		0.412	0.005	1.51 (1.13–2.02)
**2-6/ week**	315 (24.46%)		0.38	0.002	1.46 (1.14–1.87)
**Once a Day**	180 (20.52%)		0.154	0.256	1.17 (0.89–1.52)
**≥ 2 a Day**	106 (18.12%)		**Reference**		
**Smoking Current smokers**	507 (35.63%)	**<0.001**	0.656	< 0.001	1.93(1.69–2.20)
**status Former Smokers**	120 (31.83%)		0.486	< 0.001	1.63(1.29–2.04)
**Non-Smokers**	921 (22.31%)		**Reference**		
**Dental Within the past 6 months**	674 (25.97%)	0.187	-0.181	0.071	0.83(0.68–1.02)
**attendance 6 months to 1 yr.**	191 (24.21%)		-0.275	0.025	0.76 (0.60–0.97)
**1–2 yrs. ago**	165 (27.55%)		-0.101	0.431	0.9 (0.70–1.16)
**>2 yrs.**	330 (25.46%)		-0.208	0.059	0.81(0.65–1.01)
**Never**	175 (29.61%)		**Reference**		
**Diabetes Diabetic**	149 (36.34%)	**<0.001**	0.520	< 0.001	1.68(1.36–2.08)
**Normal**	1405 (25.34%)		**Reference**		

*Chi-square test was used to test significance of difference in no. participants with CPI ≥ 3.

**p-value of regression coefficient (ß).

**Table 3** reveals that age, residential location, frequency of dental visits, and history of diabetes yielded significant associations with tooth loss. Young adults had significantly lower odds of tooth loss when compared to adults aged ≥60 years (OR = 0.36; 95% CI: 0.24–0.53) and the predicted incidence of tooth loss was lower by 56.77% among adults aged 35–44 years in comparison to those aged 45–59 years. Regarding location, the predicted incidence of tooth loss was lower in rural areas than urban areas by 29.99% and lastly, diabetic participants had 3 times higher odds of tooth loss than those who were non-diabetic.

**Table 3:** Logistic regression analysis showing the associations between tooth loss and potential risk factors.

*Independent variable*	Missing Teeth > 0				
	N (%)	*p-value* *	*ß*	*p-value* **	Odds Ratio *(95% CI)*
**Age 20–34 yrs.**	177 (7.11%)	**<0.001**	-1.030	< 0.001	0.36(0.24–0.53)
**35–44 yrs.**	165 (10.79%)		-0.570	0.005	0.57(0.38–0.84)
**45–59 yrs.**	269 (15.50%)		-0.150	0.443	0.86(0.58–1.27)
**≥ 60 yrs.**	35 (17.59%)		**Reference**		
**Gender Male**	312 (10.75%)	0.818	-0.020	0.818	0.98(0.83–1.16)
**Female**	334 (10.94%)		**Reference**		
**Residence Rural**	255 (9.30%)	**<0.001**	-0.300	< 0.001	0.74(0.63–0.88)
**location Urban**	385 (12.16%)		**Reference**		
**Education Illiterate**	57 (10.61%)	0.972	-0.020	0.893	0.98(0.72–1.34)
**High school & less**	392 (10.93%)		0.010	0.899	1.01(0.84–1.21)
**2yrs. academy &**	194 (10.82%)		**Reference**		
**some college or above**					
**Teeth Never**	202 (9.62%)	**0.003**	-0.509	< 0.001	0.60 (0.46–0.79)
**brushing 2-3/ months**	49 (9.48%)		-0.525	0.006	0.59 (0.41–0.86)
**Once a week**	49 (9.37%)		-0.538	0.005	0.58 (0.40–0.85)
**2-6/ week**	144 (11.18%)		-0.341	0.019	0.71 (0.53–0.95)
**Once a Day**	108 (12.31%)		-0.232	0.134	0.79 (0.59–1.07)
**≥ 2 a Day**	88 (15.04%)		**Reference**		
**Smoking Current smokers**	166 (11.67%)	0.078	0.14	0.149	1.15 (0.95–1.39)
**status Former Smokers**	51 (13.53%)		0.31	0.051	1.36 (1.00–1.86)
**Non-Smokers**	425 (10.30%)		**Reference**		
**Dental Within the past 6 months**	327 (12.60%)	**<0.001**	1.27	<0.001	3.56(2.31–5.49)
**attendance 6 months to 1 yr.**	88 (11.15%)		1.131	< 0.001	3.10 (1.93–4.97)
**1–2 yrs. ago**	65 (10.85%)		1.101	< 0.001	3.01 (1.84–4.91)
**>2 yrs.**	134 (10.34%)		1.047	< 0.001	2.85 (1.81–4.48)
**Never**	23 (3.89%)		**Reference**		
**Diabetes Diabetic**	95 (23.17%)	**<0.001**	**1.01**	< 0.001	2.73(2.14–3.49)
**Normal**	551 (9.94%)		**Reference**		

*Chi-square test to test significant difference in tooth loss pattern

**p-value of regression coefficient (ß).

In Table 4, multivariate logistic regression done to characterize CPI≥ 3 identified poor oral hygiene practices (p < 0.001) Exp.(B) -0.014, old age (p < 0.001) Exp.(B) 0.066, low educational attainment (p < 0.001) Exp.(B) -0.033, smoking (p < 0.001) Exp.(B) 0.009 as strong predictors of periodontitis in Egyptian population.

**Table 4:** Multivariate logistic regression for periodontitis.

Independent variable	Unstandardized Coefficients		Standardized Coefficients	t	*p*-value*	95% Confidence interval for B	
	*ß*	SE	Beta			Lower	Upper
(Constant)	0.158	0.043		3.636	**< 0.001**	0.073	0.243
Age	0.066	0.007	0.138	10.008	**< 0.001**	0.053	0.079
Gender	-0.036	0.015	-0.040	-2.370	**0.018**	-0.065	-0.006
Residence Location	-0.002	0.012	-0.002	-0.168	0.866	-0.025	0.021
Education	-0.033	0.011	-0.045	-3.410	**0.002**	-0.054	-0.016
Teeth brushing	-0.014	0.003	-0.058	-4.122	**< 0.001**	-0.021	-0.008
Smoking Status	0.043	0.009	0.083	4.992	**< 0.001**	0.026	0.060
Dental attendance	0.004	0.004	0.013	0.999	0.318	-0.004	0.012
Diabetes	0.046	0.023	0.027	1.992	**0.046**	0.001	0.092

*p-value of regression coefficient (ß).

## 4. Discussion

It is incontestable that periodontal problems largely jeopardize people’s quality of life particularly in middle and old age [21]. This study is the first, in the last three decades, to report nationally representative data on occurrence of the periodontal disease and the associated determinants in the Egyptian population. Based on the findings from Egypt’s 2013–2014 national oral health survey, the odds of periodontitis in the adult population were found to be significantly higher among males, older individuals, people who were illiterate, of low educational attainment or residing in rural areas.

In the United States’ National Health and Nutrition Examination Survey 2009–2014, 42% of dentate adults aged 30 years or older suffered from periodontitis [22]. Whereas in Mainland China, data from the national survey conducted in 2015–2016 showed that 52.8–64.6% of adults aged between 35–74 years had periodontitis and the prevalence of severe periodontitis ranged from 10.6 to 43.5% [23]. The difference between the mean age of China’s national survey respondents (56.43 ± 12.40 years) and that of the current study sample (38.55 ± 11.73 years) could partially explain the higher prevalence of periodontitis among Chinese adults. On the other hand, in line with our study, there was consistency between findings of Brazil’s national oral health surveys for 2003 and 2010 in reporting higher prevalence of periodontal diseases among males despite the significant variations in their geographic distribution over time [24, 25].

Furthermore, the inverse relationship between level of education and periodontitis in this study has been verified in various populations [26, 27]. This conspicuous social gradient is commonly explained by health-related behaviors acknowledging that unfavorable oral health behaviors, such as irregular dental attendance, smoking and poor oral hygiene practices are usually more prevalent among people in disadvantaged social groups [28].

Although around 45% of all study participants reported visiting a dentist within the past 6 months, only 5.6% had healthy gums. Generally, among socially disadvantaged groups, financial constraints restrict access to oral health services to emergency care and relief of pain rather than preventive or curative dental services such as scaling especially in low middle-income countries, like Egypt, where expenditures of dental care are commonly covered by out-of-pocket payment [29]. Furthermore, at the time of our survey, 40% of the participants reported foregoing oral care despite having dental complaints. This designates that it is not uncommon that, in the Egyptian society, people might not seek periodontal treatment even when they experience symptoms, and this is more likely to happen among less educated and economically impoverished groups.

On the other hand, according to the WHO health observatory, Egypt’s dentist/population (D/P) ratio has increased only from 1.1/10,000 in 2004 to 1.632/10,000 in 2014 and no data existed on any dental auxiliaries delivering preventive and/or basic curative oral care [30]. Usually, rural areas lack dental workforce probably due to lower remuneration [31] which could partially explain the limited availability of dental services including periodontal treatment and thus increased burden of periodontal problems among rural Egyptians.

Tobacco consumption in any form has been strongly linked to extensive periodontal destruction and eventually tooth loss particularly in males [32], and recently, the American Academy of Periodontology has identified smoking as a modifier for severity of periodontitis [33]. In line, our findings indicate that periodontal health status tends to be significantly worse among both current and former smokers and smoking was found to be one of the strongest predictive factors for periodontitis among Egyptian adults. According to Egypt’s Demographic Health Survey (DHS) 2015, 46% of males aged 15–59 years reported using various tobacco products compared to 0.2% of females and this percentage increased with age peaking at 59% in the 50–54 age group [34]. This might also explain the significant increase in prevalence of periodontitis from 18% among young adults aged (20–34 years) to 35% and 38% in adults aged (45–59 years) and the elderly ≥60 years respectively.

Despite the strong evidence on the bidirectional association between diabetes and periodontal disease [35, 36], yet, to date, this relationship is still controversial [37]. Among Egyptian adults, however, diabetic patients were found to be significantly more liable to suffer from both periodontitis and tooth loss which further corroborates the findings of Costa et al [38].

Low SES is considered by far the most consistent predictive factor for tooth loss [26], yet, in our study no significant socioeconomic difference was detected with respect to prevalence of tooth loss among Egyptian adults. Similarly, the socioeconomic position failed to describe people’s oral health status in other populations where some social classes exhibited poorer oral health and significantly more tooth loss when compared to lower classes [39]. One plausible explanation for this inconsistency is that generally, socioeconomic position is determined based on individual’s income, level of educational attainment, and occupational class and thus understanding how it impacts health outcomes is quite complex [40]. Nevertheless, in the current study, only level of education was considered as a measure of SES ignoring the impact of the inter-section between various social indicators on health outcomes as implied by the concept of examining health inequalities using the intersectionality approach [41].

On the other hand, the odds of tooth loss were significantly higher among participants who attended dental facilities when compared to those who never visited a dentist. This suggests that, particularly in communities with low SES, people would have diseased teeth extracted rather than retained when more conservative treatment options are unavailable or unaffordable.

According to Kailembo et al [42], a strong association exists between edentulism and old age and people living in rural communities are more likely to have all their teeth removed. In the present study, at the time of data collection, edentulous individuals were excluded from the overall sample as done in other national surveys [43] and as the analysis showed, out of 5954 participants, only 199 (3.34%) were aged ≥60 years. Thus, the severity of tooth loss might have been underestimated which might explain the higher odds of tooth loss in urban rather than rural areas where people are more likely to be “totally edentulous”. Furthermore, only data on “teeth missing due to reasons other than dental caries” was analyzed, which might, as well, have underestimated tooth loss in this study. It is also possible that due to recall bias participants might have over-reported dental caries as the reason for tooth extraction as people presumably link dental pain to tooth cavitation, although both dental caries and periodontal disease have been proven to be main causes for tooth mortality [44].

Gingival bleeding and calculus formation are not indicators of periodontitis, however, their prevalence was included in our study analysis because they are considered warning signs of possible later development of periodontal problems, if not restrained. In the studied sample, it was found that the prevalence of gingival bleeding and calculus formation were 6.7% and 62% respectively with greater preponderance among females and young adults.

Notably, owing to altered immune and hormonal status the response of gingival tissues to plaque is usually aggravated especially during the second and third trimester of gestation [45]. Moreover, there is strong evidence that pregnant women do not seek dental care during pregnancy partly due to lack of knowledge of association between poor oral health and adverse pregnancy outcomes [46]. Consequently, it is conceivable that underutilization of oral care, poor oral hygiene practices along with high fertility rates reported in Egypt [47] might collectively elucidate the high prevalence of gingivitis among Egyptian females particularly in low socioeconomic groups.

One limitation of the current study was the cross-sectional design which inherently does not exhibit the temporal relationship between studied variables, and thus a causal relationship between periodontitis and the potential risk factors could not be concluded. It is also noteworthy that relying on self-reporting in collecting data on health-related behaviors might have introduced social desirability bias as when participants tend to overreport good health-related behaviors such as oral hygiene practices and frequency of dental visits or underreport undesirable behaviors such as smoking among females. A response bias might have also occurred if participants tended to respond systematically to questionnaire items on certain basis other than the specific content of each item [48, 49].

Though using only pocket depths to assess periodontitis might have underestimated disease prevalence yet combining clinical attachment loss (CAL) with periodontal pockets to assess periodontal diseases is known to be debatable, acknowledging that in different age groups, the same level of pocket depth or CAL might have different implications with respect to progression of periodontal disease and tooth loss [50]. Furthermore, periodontal pockets are considered reliable indicators of presence of active disease process, on the other hand CAL indicates irreversible periodontal destruction and might persist following successful periodontal therapy or in the presence of gingival recession and thus depending only on attachment loss to assess disease progress could be of moderate or low success [51, 52].

Among strengths of this study is the national representation of the survey owing to the large sample size, random selection of large number of gathering points and thus coverage of all geographic areas as well as the diversity in age groups which collectively verify generalizing the study findings to the Egyptian adult population. Though, in the current study, only level of educational attainment was used as a measure of SES however education was identified, since decades, as a strong determinant of an individual’s future employment and subsequent income [53]. Additionally, adopting the WHO basic standardized methods for oral health surveys in reporting information on oral diseases facilitates both surveillance of intra-country disease trends and inter-country comparisons [17].

In conclusion, the current study findings demonstrate the heavy burden of periodontal diseases in the Egyptian adult population and provides necessary information for undertaking population-based action to control this public health issue at national level. Because periodontal diseases could exacerbate various systemic conditions, adoption of a common risk factor approach for addressing modifiable risk factors shared with other non-communicable diseases (NCD) provides a cost-effective public health strategy to reduce oral disease burden in the Egyptian population and achieve NCD-related targets of the 2030 Sustainable development goals.

## Supporting information

S1 Checklist STROBE (Strengthening The Reporting of OBservational Studies in Epidemiology) checklist. (PDF)

S1 Dataset Dataset for 60 pats CPI. (XLSX)

## References

[1] Baehni PC. Translating science into action—prevention of periodontal disease at patient level. Periodontol 2000, 2012. 60(1): p. 162–72. doi: 10.1111/j.1600-0757.2011.00428.x 22909114

[2] Kassebaum NJ, et al. Global, Regional, and National Prevalence, Incidence, and Disability-Adjusted Life Years for Oral Conditions for 195 Countries, 1990–2015: A Systematic Analysis for the Global Burden of Diseases, Injuries, and Risk Factors. J Dent Res, 2017. 96(4): p. 380–387. doi: 10.1177/0022034517693566 28792274 PMC5912207

[3] Global, regional, and national disability-adjusted life-years (DALYs) for 333 diseases and injuries and healthy life expectancy (HALE) for 195 countries and territories, 1990–2016: a systematic analysis for the Global Burden of Disease Study 2016. Lancet, 2017. 390(10100): p. 1260–1344. doi: 10.1016/S0140-6736(17)32130-X 28919118 PMC5605707

[4] Thomson WM, Sheiham A, Spencer AJ. Sociobehavioral aspects of periodontal disease. Periodontol 2000, 2012. 60(1): p. 54–63. doi: 10.1111/j.1600-0757.2011.00405.x 22909106

[5] Persson GR. Perspectives on periodontal risk factors. J Int Acad Periodontol, 2008. 10(3): p. 71–80. 18714932

[6] Tonetti MS, et al. Dental caries and periodontal diseases in the ageing population: call to action to protect and enhance oral health and well-being as an essential component of healthy ageing—Consensus report of group 4 of the joint EFP/ORCA workshop on the boundaries between caries and periodontal diseases. J Clin Periodontol, 2017. 44 Suppl 18: p. S135–s144. doi: 10.1111/jcpe.12681 28266112

[7] Chapple IL, et al. Primary prevention of periodontitis: managing gingivitis. J Clin Periodontol, 2015. 42 Suppl 16: p. S71–6. doi: 10.1111/jcpe.12366 25639826

[8] Petersen PE, Ogawa H. The global burden of periodontal disease: towards integration with chronic disease prevention and control. Periodontol 2000, 2012. 60(1): p. 15–39. doi: 10.1111/j.1600-0757.2011.00425.x 22909104

[9] Jin LJ, et al. Global oral health inequalities: task group—periodontal disease. Adv Dent Res, 2011. 23(2): p. 221–6. doi: 10.1177/0022034511402080 21490234

[10] Enwonwu CO and Salako N. The periodontal disease-systemic health-infectious disease axis in developing countries. Periodontol 2000, 2012. 60(1): p. 64–77. doi: 10.1111/j.1600-0757.2012.00447.x 22909107

[11] Petersen PE, Baehni PC. Periodontal health and global public health. Periodontol 2000, 2012. 60(1): p. 7–14. doi: 10.1111/j.1600-0757.2012.00452.x 22909103

[12] Tonetti MS, et al. Impact of the global burden of periodontal diseases on health, nutrition and wellbeing of mankind: A call for global action. J Clin Periodontol, 2017. 44(5): p. 456–462. doi: 10.1111/jcpe.12732 28419559

[13] Central Agency for Public Mobilization and Statistics. Egypt Statistical Yearbook 2017. Accessible from: http://ghdx.healthdata.org/organizations/central-agency-public-mobilization-and-statistics-capmas-egypt.

[14] Abbass MMS, et al. The occurrence of periodontal diseases and its correlation with different risk factors among a convenient sample of adult Egyptian population: a cross-sectional study. F1000Res, 2019. 8: p. 1740. doi: 10.12688/f1000research.20310.2 32494356 PMC7236581

[15] Hegab M, Alnawawy M. The Prevalence of gingival recession in the Egyptian Population. Perio J. 2020; 4(1), 1–10. 10.26810/perioj.2020.a1.

[16] World Health Organization, Regional office for the Eastern Mediterranean. Egypt releases results of epidemiological study on oral health status. 2014. http://www.emro.who.int/egy/egypt-events/results-of-epidemiological-study-on-oral-health-status-released.html Accessed April 20, 2020.

[17] Petersen PE, Baez RJ, World Health Organization. Oral Health Surveys: Basic Methods. 5^th^ ed. Geneva, Switzerland, 2013.

[18] Ainamo J, et al. Development of the World Health Organization (WHO) community periodontal index of treatment needs (CPITN). Int Dent J, 1982. 32(3): p. 281–91. 6958657

[19] McHugh ML. Interrater reliability: the kappa statistic. Biochem Med (Zagreb), 2012. 22(3): p. 276–82. 23092060 PMC3900052

[20] Ursachi G, Horodnic IA, Zait A. How Reliable are Measurement Scales? External Factors with Indirect Influence on Reliability Estimators. Procedia Economics and Finance, 2015. 20: p. 679–686.

[21] Ferreira MC, et al. Impact of periodontal disease on quality of life: a systematic review. J Periodontal Res, 2017. 52(4): p. 651–665. doi: 10.1111/jre.12436 28177120

[22] Eke PI, et al. Periodontitis in US Adults: National Health and Nutrition Examination Survey 2009–2014. J Am Dent Assoc, 2018. 149(7): p. 576–588.e6. doi: 10.1016/j.adaj.2018.04.023 29957185 PMC8094373

[23] Jiao J, et al. The prevalence and severity of periodontal disease in Mainland China: Data from the Fourth National Oral Health Survey (2015–2016). J Clin Periodontol, 2021. 48(2): p. 168–179. 33103285 10.1111/jcpe.13396

[24] SB 2000. *Condições de saúde bucal da população brasileira 2002–2003*, *Resultados principais*. Brasília-DF: Ministério da Saúde, Secretaria de Atenção à Saúde, Departamento de Atenção Básica, Coordenação Nacional de Saúde Bucal. 2004.

[25] Vettore MV, Marques RA, Peres MA. [Social inequalities and periodontal disease: multilevel approach in SBBrasil 2010 survey]. Rev Saude Publica, 2013. 47 Suppl 3: p. 29–39. doi: 10.1590/s0034-8910.2013047004422 24626579

[26] Buchwald S, et al. Tooth loss and periodontitis by socio-economic status and inflammation in a longitudinal population-based study. J Clin Periodontol, 2013. 40(3): p. 203–11. doi: 10.1111/jcpe.12056 23379538

[27] Kocher T, et al. Risk determinants of periodontal disease—an analysis of the Study of Health in Pomerania (SHIP 0). J Clin Periodontol, 2005. 32(1): p. 59–67. doi: 10.1111/j.1600-051X.2004.00629.x 15642060

[28] Huijts T, et al. Educational inequalities in risky health behaviours in 21 European countries: findings from the European social survey (2014) special module on the social determinants of health. Eur J Public Health, 2017. 27(suppl_1): p. 63–72. doi: 10.1093/eurpub/ckw220 28355636

[29] Bernabé E, Masood M, Vujicic M. The impact of out-of-pocket payments for dental care on household finances in low and middle income countries. BMC Public Health, 2017. 17(1): p. 109. doi: 10.1186/s12889-017-4042-0 28114967 PMC5260123

[30] WHO Global Health Observatory Data Repository. Dentistry personnel. Updated 2018. https://apps.who.int/gho/data/node.main.HWF2. Accessed May 24, 2020.

[31] Voinea-Griffin A, Solomon ES. Dentist shortage: an analysis of dentists, practices, and populations in the underserved areas. J Public Health Dent, 2016. 76(4): p. 314–319. doi: 10.1111/jphd.12157 27198620

[32] Bergström J. Cigarette smoking as risk factor in chronic periodontal disease. Community Dent Oral Epidemiol, 1989. 17(5): p. 245–7. doi: 10.1111/j.1600-0528.1989.tb00626.x 2791514

[33] Tonetti MS, Greenwell H, Kornman KS. Staging and grading of periodontitis: Framework and proposal of a new classification and case definition. Journal of Periodontology, 2018. 89(S1): p. S159–S172. 29926952 10.1002/JPER.18-0006

[34] Ministry of Health and Population, Cairo, Egypt. El-Zanaty and Associates. Health Issues Survey, 2015. The DHS Program ICF International. Rockville, Maryland, USA; 2015. https://dhsprogram.com/publications/publication-fr313-dhs-final-reports.cfm Accessed, May 5^th^, 2020.

[35] Chávarry NG, et al. The relationship between diabetes mellitus and destructive periodontal disease: a meta-analysis. Oral Health Prev Dent, 2009. 7(2): p. 107–27. 19583037

[36] Kocher T, et al. Periodontal complications of hyperglycemia/diabetes mellitus: Epidemiologic complexity and clinical challenge. Periodontol 2000, 2018. 78(1): p. 59–97. doi: 10.1111/prd.12235 30198134

[37] Joshipura KJ, et al. Longitudinal association between periodontitis and development of diabetes. Diabetes Res Clin Pract, 2018. 141: p. 284–293. doi: 10.1016/j.diabres.2018.04.028 29679620 PMC6016543

[38] Costa FO, et al. Progression of periodontitis and tooth loss associated with glycemic control in individuals undergoing periodontal maintenance therapy: a 5-year follow-up study. J Periodontol, 2013. 84(5): p. 595–605. 22769441 10.1902/jop.2012.120255

[39] Novrinda H, et al. Neo-Marxian social class inequalities in oral health among the South Korean population. Community Dent Oral Epidemiol, 2019. 47(2): p. 162–170. doi: 10.1111/cdoe.12439 30548668

[40] Muntaner C, et al. Social class and mental health: testing exploitation as a relational determinant of depression. Int J Health Serv, 2015. 45(2): p. 265–84. doi: 10.1177/0020731414568508 25813501 PMC4747250

[41] Evans CR, et al. A multilevel approach to modeling health inequalities at the intersection of multiple social identities. Soc Sci Med, 2018. 203: p. 64–73. doi: 10.1016/j.socscimed.2017.11.011 29199054

[42] Kailembo A, Preet R, Stewart Williams J. Common risk factors and edentulism in adults, aged 50 years and over, in China, Ghana, India and South Africa: results from the WHO Study on global AGEing and adult health (SAGE). BMC Oral Health, 2016. 17(1): p. 29. doi: 10.1186/s12903-016-0256-2 27465011 PMC4964081

[43] Dalazen CE, et al. Contextual and Individual Factors Influencing Periodontal Treatment Needs by Elderly Brazilians: A Multilevel Analysis. PLoS One, 2016. 11(6): p. e0156231. doi: 10.1371/journal.pone.0156231 27249677 PMC4889082

[44] Aida J, et al. Reasons for permanent tooth extractions in Japan. J Epidemiol, 2006. 16(5): p. 214–9. doi: 10.2188/jea.16.214 16951541 PMC7683702

[45] Suresh L, Radfar L. Pregnancy and lactation. Oral Surg Oral Med Oral Pathol Oral Radiol Endod, 2004. 97(6): p. 672–82. doi: 10.1016/j.tripleo.2004.02.002 15184848

[46] Saddki N, Yusoff A, Hwang YL. Factors associated with dental visit and barriers to utilisation of oral health care services in a sample of antenatal mothers in Hospital Universiti Sains Malaysia. BMC Public Health, 2010. 10: p. 75. doi: 10.1186/1471-2458-10-75 20163741 PMC2834630

[47] Sayed H. Trends of fertility levels in Egypt in recent years. United Nations Population Fund; 2019.

[48] Demetriou C, Ozer BU, Essau C. Self-report questionnaires. In: Cautin R, Lilienfeld S. The Encyclopedia of Clinical Psychology. Malden, MA: John Wiley & Sons, Inc. 2015.

[49] Paulhus DL. Measurement and control of response bias. Measures of Personality and Social Psychological Attitudes, 1991; 1, 17–59.

[50] Papapanou PN, Susin C. Periodontitis epidemiology: is periodontitis under-recognized, over-diagnosed, or both? Periodontol 2000, 2017. 75(1): p. 45–51. doi: 10.1111/prd.12200 28758302

[51] Burt B, Eklund S. Dentistry, Dental Practice, and the Community. 5th ed. Philadelphia, Pa: WB Saunders Co; 1999.

[52] Beck JD, Koch GG, Offenbacher S. Attachment loss trends over 3 years in community-dwelling older adults. J Periodontol, 1994. 65(8): p. 737–43. doi: 10.1902/jop.1994.65.8.737 7965548

[53] Lynch JW, and Kaplan GA. Socioeconomic position. In: Berkman LF, Kawachi I (eds), Social Epidemiology. 1st ed. Oxford: Oxford University Press; 2000. p. 13–35.

